# Description and distribution of *Tuberincognitum* sp. nov. and *Tuberanniae* in the Transmexican Volcanic Belt

**DOI:** 10.3897/mycokeys.41.28130

**Published:** 2018-10-11

**Authors:** Carolina Piña Páez, Gregory M. Bonito, Gonzalo Guevara-Guerrero, Michael A. Castellano, Roberto Garibay-Orijel, James M. Trappe, Rafael Peña Rámirez

**Affiliations:** 1 Instituto de Biología, Universidad Nacional Autónoma de México, Tercer Circuito s/n, Ciudad Universitaria Delegación Coyoacán, A.P. 70-233, C.P. 04510 Ciudad de México, México; 2 Department of Plant, Soil and Microbial Sciences, Michigan State University, East Lansing, MI 48824, USA; 3 Instituto Tecnológico de Ciudad Victoria. Av. Portes Gil 1301 Poniente, 87010 Ciudad Victoria, Tamaulipas, México; 4 US Department of Agriculture, Forest Service, Northern Research Station, Forestry Sciences Laboratory, Corvallis, Oregon, 97331, USA

**Keywords:** Sequestrate fungi, truffles, Ascomycota, Systematics, new species

## Abstract

The genus *Tuber* is a lineage of diverse ectomycorrhizal, hypogeous, sequestrate ascomycete fungi that are native to temperate forests in the Northern Hemisphere. Recently, many new species of *Tuber* have been described in North America and Asia, based on morphological characteristics and molecular data. Here we describe and illustrate a new species, *Tuberincognitum*, based upon phylogenetic analysis and morphological description. We also present a new record for *Tuberanniae* in México. These two *Tuber* species are distributed in the Transmexican Volcanic Belt in the states of México, Michoacán, Guanajuato, Querétaro and Tlaxcala at altitudes between 2,000 and 3,200 meters. These species are associated with *Pinus* (*T.anniae*) and *Quercus* forests (*T.incognitum*).

## Introduction

Fungal species, within the genus *Tuber*, produce hypogeous, sequestrate ascomata, that are more commonly known as truffles. These fungi are ectomycorrhizal (EcM) symbionts of angiosperm or gymnosperm hosts, including many species of trees as well as orchids. Plant hosts provide their EcM symbionts with carbohydrates in exchange for greater access to water and nutrients (Wurzburger et al. 2001; Bidartondo et al. 2004; Walker et al. 2005; Shefferson et al. 2008). The genus *Tuber* has been studied intensively over the past century, largely due to its economic importance as an edible fungus. Most of these efforts have been directed towards European species with economic value (e.g. *Tubermelanosporum*, *Tubermagnatum*, *Tuberaestivum*), which reside in a few clades, neglecting most of the diversity in this genus. Reference and environmental sequences data were recently used to infer a minimum of 180–230 species of *Tuber* worldwide (Bonito et al. 2010) and substantiate that most *Tuber* diversity resides within less studied and non-economically important lineages delimitated as the Rufum, Puberulum and Maculatum clades. In México, eighteen *Tuber* species are known and have been formally described. The majority of the collections of these described species are from northeast and central México. In this study, we propose the new species *Tuberincognitum* and provide the first report of *T.anniae* in México based on morphological characteristics and phylogenetic analyses.

## Materials and methods

### Morphological observation

Ascomata were collected from the states of Guanajuato, México, Michoacán, Querétaro, Tlaxcala and were deposited in herbaria at Oregon State University (OSC), Herbario Nacional de México (MEXU) and Herbario José Castillo Tovar (ITCV). Macroscopic characters were recorded from fresh specimens and microscopic characters were described from both sections of fresh specimens and dried specimens mounted in 5% potassium hydroxide (KOH) following protocols from Castellano et al. (1989).

### DNA sequencing and phylogenetic analyses

DNA was extracted from ascomata of collections OSC157842 and OSC150066 using a CTAB chloroform extract protocol and ITS rDNA was amplified and sequenced as previously described (Bonito et al. 2010). Tissue samples from collections MEXU 26504, MEXU 26541, MEXU 26218 and MEXU25995 were sent to the Canadian Center of Barcoding (CCDB) for extraction, amplification, sequencing and barcoding of the Internal Transcribed Spacers (ITS). The ITS region was amplified with ITS1f and ITS4 primers (White et al. 1990). The sequences were edited in Geneious 7.1 (http://www.geneious.com, Kearse et al. 2012). The distribution and ecology of these species was complemented with soil DNA data from central and south México through a BLASTn search against the Mexican Soil Fungi Database in Geneious 7.1. This database includes ITS2 sequences of soil fungi (total DNA soil extractions) from central and southern México as part of an ongoing project, which has been partially published by Argüelles-Moyao and Garibay-Orijel (2018).

DNA sequences were manually trimmed and edited with Sequencher 4.0 (Gene Codes Corp., Ann Arbor, Michigan). ITS sequences were queried against the NCBI public database GenBank by use of the BLASTn algorithm to retrieve similar sequences (Altschul et al. 1990). Collated DNA sequences were aligned with MUSCLE in Mesquite 3.04 (Maddison and Maddison 2015; Edgar 2004). Ambiguously aligned regions were excluded from the alignment. Phylogenetic analyses were conducted on ITS rDNA alignments through the CIPRES portal (www.phylo.org, Miller et al. 2010). Maximum Likelihood (ML) searches were conducted with RAxML v.7.2.8 using rapid bootstrapping of 1,000 pseudoreplicates (Stamatakis 2014). Bayesian Inference (BI) was carried out with MrBayes v3.2.6 (Ronquist and Huelsenbeck 2003). To estimate posterior probabilities, 20,000,000 Markov chain Monte Carlo (MCMC) simulation generations were run in two parallel searches on four chains, with trees sampled every 1,000^th^ generation, with the first 5,000 trees discarded as burn-in (convergence of parallel runs). Bootstrap support, based on 1,000 iterations, was considered informative where it was ≥ 70% and posterior probability was considered significant where it was ≥ 99%. Sequences, generated in this study, are available on GenBank under the accession numbers GQ221447, KC152267, KC152256, KJ595013, KJ595014 and MH174661 (Table 1) and in the BOLD systems database (www.barcodinglife.org).

**Table 1.** Accession and voucher numbers of sequences generated in this paper.

Taxon	GenBank	Voucher	Origin	Reference
***T.incognitum*** Piña Páez. Bonito, Guevara & Castellano	GQ221447	OSC 150066	Ascoma	This paper
	KJ595013	MEXU 26218	Ascoma	This paper
	KJ595014	MEXU 25995	Ascoma	This paper
	MH174661	–	EcM	This paper
	MH447961	ITCV 1695	Ascoma	This paper
*T.anniae* W. Colgan & Trappe	MH174660	OSC 157842	Ascoma	This paper
*T.bonitoi* G. Guevara & Trappe	KC152256	MEXU 26541	Ascoma	Guevara et al. 2015
*Tuber* sp. 3	KJ152267	MEXU 26504	Ascoma	This paper

## Results

### Descriptions

#### Tuber incognitum

Taxon classificationFungiPezizalesTuberaceae

Piña Páez, Bonito, Guevara & Castellano sp. nov.

MB824931

Fig. 1a–d

##### Type.

MÉXICO, State of Querétaro, Huimilpan, San Pedro, under *Quercuscrassifolia* Humb. and Bonpl., *Quercus* spp., hypogeous, gregarious, 24 September 1996, M.A. Castellano (Holotype: OSC 150066), GB GQ221447. State of Michoacán, Zinapécuaro, el Jaral, under *Quercuspolymorpha* Schltdl. and Cham., hypogeous, solitary or in groups of two, 2380 m alt., 19°46'48"N, -100°47'24"W, 4 September 2008, R. Garibay-Orijel (Paratype: MEXU 25995), GB KJ595014. State of México, Temascaltepec, under *Quercus* spp., hypogeous, solitary, 2011 m alt., 19°04'12"N, -100°01'48"W, 8 July 2009, R. Garibay-Orijel (Paratype: MEXU 26218), GB KJ595013.

##### Diagnosis.

*Tuberincognitum* is distinctive in the structure of its peridium (two-layered) and spore size (25–55 × 20–44 μm), which separates it from the rest of the species within the Puberulum clade reported from México.

##### Etymology.

Incognitum is Latin for unknown. The name incognitum is not derived from its morphology, rather from the fact that it was overlooked for so long. The holotype was collected in 1996 and not described until now.

##### Description.

***Ascomata*** 10–15 mm broad, subglobose to slightly irregular, white with light brown areas when dry, glabrous, with canals that continue with the veins into the gleba. Gleba pinkish to purplish pale-brown in youth, dark brown at maturity, marbled with white veins. Odour fruity, pleasant.

***Peridium*** two-layered, when handled the upper layer is lost and only the inner layer is observable under the light microscope, 350–400 μm thick, pellis 175–240 μm thick, composed of isodiametric or angular cells, 6–15 μm broad, walls 1.75–2.0 µm thick, yellowish hyaline in KOH. Subpellis 110–140 µm thick, composed of septate, interwoven hyphae (*textura intricata*), 4.5–7.0 µm broad, thin walled < 1 μm thick, hyaline in KOH. Gleba composed of septate, interwoven hyphae (*textura epidermoidea*), 5.0–7.5 µm broad, thin walled < 1 μm thick, hyaline in KOH. ***Ascospores*** broadly ellipsoid; excluding their alveolate-reticulate ornamentation, in 1-spored asci 45–55 × 34–44 μm (Q = 1.3), 2-spored 37–43 × 29–34 μm (Q = 1.25–1.36), 3-spored 30–42 × 26–31 μm (Q = 1.2–1.37), 4-spored 28–33 × 24–28 μm (Q = 1.09–1.25) and 5-spored 25–28 × 20–28 μm (Q = 1.2–1.25), spore colour orange-yellow in KOH, the walls > 2 μm thick; reticulum with 3–8 alveolae across the spore surface; the alveolar walls 3.5–4.0 μm tall. ***Asci*** globose, subglobose to broadly ellipsoid, pyriform, 88–100 × 70–95 μm, pedicel lacking to prominent, hyaline in KOH, hyphae around the asci prostrated or interwoven, cylindrical, 3.5–6.0 μm broad at the septa, thin walled, hyaline in KOH.

**Figure 1.**
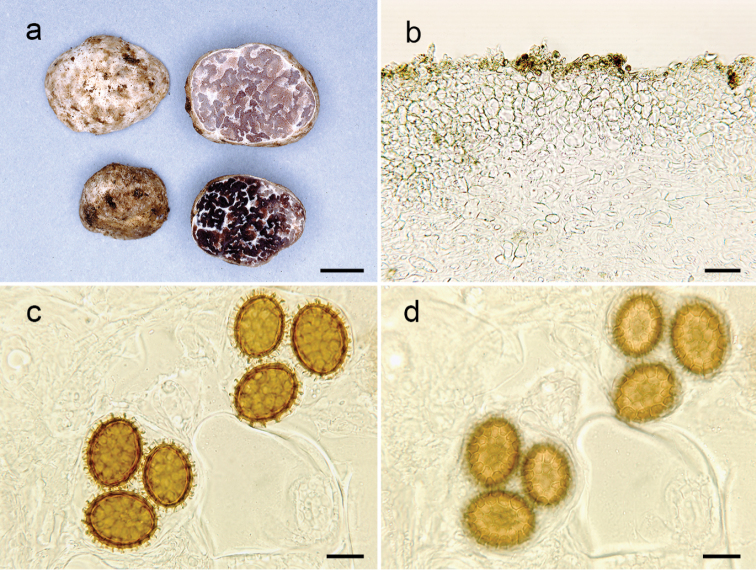
*Tuberincognitum* (Holotype, OSC 150066). **a** Ascoma, surface and cross-section view **b**Peridium in cross-section **c** Light microscopy of spores in cross-sectional view, highlighting the spines and ornamentation **d** Light microscopy of spores in surface view, highlighting the surface and reticulum. Scale bars: 5 mm (**a**), 20 µm (**b**), 15 µm (**c, d**).

##### Distribution and ecology.

Only known from central and southwest México (Querétaro, Michoacán, State of México, Guanajuato and Hidalgo). Ascocarps always associated with *Quercus* species (*Q.crassifolia*, *Q.polymorpha*). An EcM association with *Quercus* has been verified (MH174661) and its DNA has been recovered only from soil in *Quercus* forest in Hidalgo, México.

##### Additional collections examined.

MÉXICO, State of Guanajuato, Guanajuato, Las Palomas, under *Quercus* spp., hypogeous, in groups of two, 2534 m alt., 21°03'50"N, -101°13'23"W, 10 October 2016, R. Peña-Ramirez (ITCV 1695).

##### Taxonomic comments.

*Tuberincognitum* resembles *Tuberpseudoseparans* in the colour of the peridium and the lack of dermatocystidia but differs by the size of the spores (being smaller in *T.incognitum*, 31–50 × 24–37 μm vs. *T.pseudoseparans*, 46–65 × 34–46 μm) and in the thickness of the peridium (being thinner in *T.pseudoseparans*, by ± 250 μm). *Tuberincognitum* is similar to *Tuberbonitoi* in spore size and ornamentation, but differs by the presence of dermatocystidia, which are absent in *T.incognitum* and the thickness of the peridium, being thicker in *T.bonitoi* (200–500 μm). *Tuberincognitum* is similar to *Tuberguzmanii* in the peridial organisation, both species have a well differentiated two-layered peridium but differ in the thickness (being thinner in *T.guzmanii*, 100–160 μm) and spore ornamentation (alveolate reticulum, 2–4 μm tall) and the size of the spores (being larger in *T.guzmanii*, 27–68 × 30–50 μm). The collection from Guanajuato represents a young developmental stage of *T.incognitum*, this collection has a thinner peridium (130–345 μm) and smaller spores (1-spored asci 23–35 × 19–25 μm, Q = 1.09 1.59; 2-spored 18–29 × 17–22 μm, Q = 1.0–1.61; 3-spored 30–42 × 26–31 μm, Q = 1.11–1.2; 4-spored 23–27 × 19–25 μm, Q = 1.08–1.26). These differences represent morphological variation within the species and its identity was confirmed with molecular data.

#### Tuber anniae

Taxon classificationFungiPezizalesTuberaceae

W. Colgan & Trappe

Fig. 2a–d

##### Description.

***Ascomata*** subglobose to slightly irregular, 10–12 mm broad, white, cream, light brown when dry. Peridium thin, < 0.2 mm, smooth to velvety, irregularly roughened, furrows with depressions continuing as canals into the gleba. Gleba solid, brown, marbled with white veins that emerge as depressions on the peridium. Odour and taste not recorded.

***Peridium*** 85–140 μm thick; pellis a pseudoparenchyma, 40–65 μm thick, cells 6–18 μm broad, versiform, isodiametric, squared, rectangular or angular, hyaline to yellowish in KOH, thick walled (> 1.0 μm), dermatocystidia absent; subpellis 45–75 μm thick, of hyaline, septate, interwoven hyphae (*textura epidermoidea*), 4.0–5.5 µm broad, thin walled, < 1 μm thick. Gleba of hyaline, interwoven, sinuous hyphae, 5.0–7.5 µm broad, constrained at the septum, 3.0–4.5 μm broad at the septa, thin-walled (< 1.0 μm).

***Ascospores*** subglobose; excluding their alveolate-reticulate ornamentation, 1-spored asci 40–50 × 30–46 μm (Q= 1.03–1.15), 2-spored 28–38 × 26–35 μm (Q = 1.05–1.13), 3-spored 26–33 × 24–30 μm (Q = 1.04–1.15), spore colour orange-yellowish in KOH; walls > 2 μm thick, yellow; reticulum with 5–6 aveolae across the spore surface; the alveolar walls 3–4.5 μm tall. ***Asci*** subglobose, 84–105 × 75–85 μm, pedicel lacking to prominent, walls with 2–3 layers, hyaline in KOH; hyphae around the asci interwoven, 3.5–5.5 μm broad at the septum, thin walled (< 1.0 μm), hyaline in KOH.

**Figure 2.**
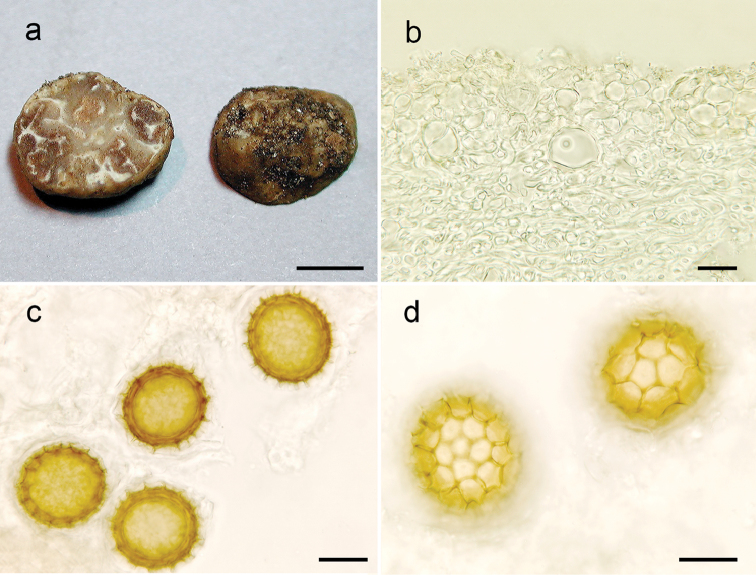
*Tuberanniae* (OSC 157842). **a** Ascoma, surface and cross-section view **b**Peridium in cross-section **c** Light microscopy of spores in cross-sectional view, highlighting the spines and ornamentation **d** Light microscopy of spores in surface view, highlighting the surface and reticulum. Scale bars: 5 mm (**a**), 15 µm (**b, c, d**).

##### Distribution and ecology.

Wang et al. (2013) reported from Europe; Colgan and Trappe 1997; Bonito et al. (2010) reported in North America. Here, we extended the distribution to central México (State of México and Tlaxcala). In Finland, *T.anniae* has been confirmed to establish association with *P.sylvestris* L. (Wang et al. 2013). In Washington, this species has been confirmed to establish association with *Pseudotsugamenziesii* (Mirbel) Franco (Bonito et al. 2010). In México, sporocarps always collected co-occurring with *Pinusleiophylla* Schiede and Deppe and *Abiesreligiosa* (Kuntch) Schldl. and Cham. In México, the only environmental DNA of this species has been recovered from soil in conifer forests in Tlaxcala associated with *Pinusmontezumae* and in State of México associated with *A.religiosa* (Argüelles-Moyao and Garibay-Orijel 2018).

##### Collections examined.

MÉXICO, State of Tlaxcala, Huamantla, cañada central, La Malinche National Park, under *Pinusleiophylla* Schiede and Deppe and *Abiesreligiosa* (Kunth) Schltdl. and Cham., hypogeous, solitary, 3220 m alt., 19°14'7"N, -97°59'9"W, 23 September 2007, G.M. Bonito (OSC 157842), GB MH174660.

##### Taxonomic comments.

*Tuberanniae* is similar to *Tuberpacificum* Trappe, Castellano and Bushnell, however, the latter species has narrower, ellipsoid spores (23–15 × 16–35 μm) and a thicker peridium (250–400 μm) than the former. *T.pacificum* has also been found co-occurring with *Pseudotsugamenziesii* and *Tsugaheterophylla* (Raf.) Sarg. along costal Oregon, while *T.anniae* has been found co-occurring with *P.leiophylla* and *A.religiosa*.

*Tuberanniae* was first described by Colgan and Trappe (1997). The holotype (from Washington) and the other collections reported were from the Pacific Northwest in the US and reported co-occurring with *P.menziesii*. The *T.anniae* complex of species has been proposed based on phylogenetic analysis using ITS region (Wang et al. 2013). The collection from México is very similar to the holotype collection, however, the latter has a brown to dark olive-brown peridium and its spores have thicker (up to 5 μm) spore walls than the former. Additionally, *T.anniae*, as described by Colgan and Trappe (1997), has mostly globose spores with 10–16 alveolae across the spores. The Finnish collections exhibit subtle morphological differences in comparison with the collections from North America. The Finnish specimens have a smooth peridial surface, except along the grooves and around the pits (Wang et al. 2013), while the holotype specimen was reported to be smooth and lack dermatocystidia (Colgan and Trappe 1997). It seems that the presence of dermatocystida only along the grooves and/or at the bottom of pits in *Tuber* collections is likely the result of handling of the ascoma during processing (Dr. D. Luoma, personal communication). Additionally, the spores from the Finnish specimens have larger dimensions (27–60 × 27–56 μm; Q = (1.00) 1.05–1.20 (1.33)) than the specimens described for *T.anniae* by Colgan and Trappe from Washington (1997).

## Discussion

**Figure 3.**
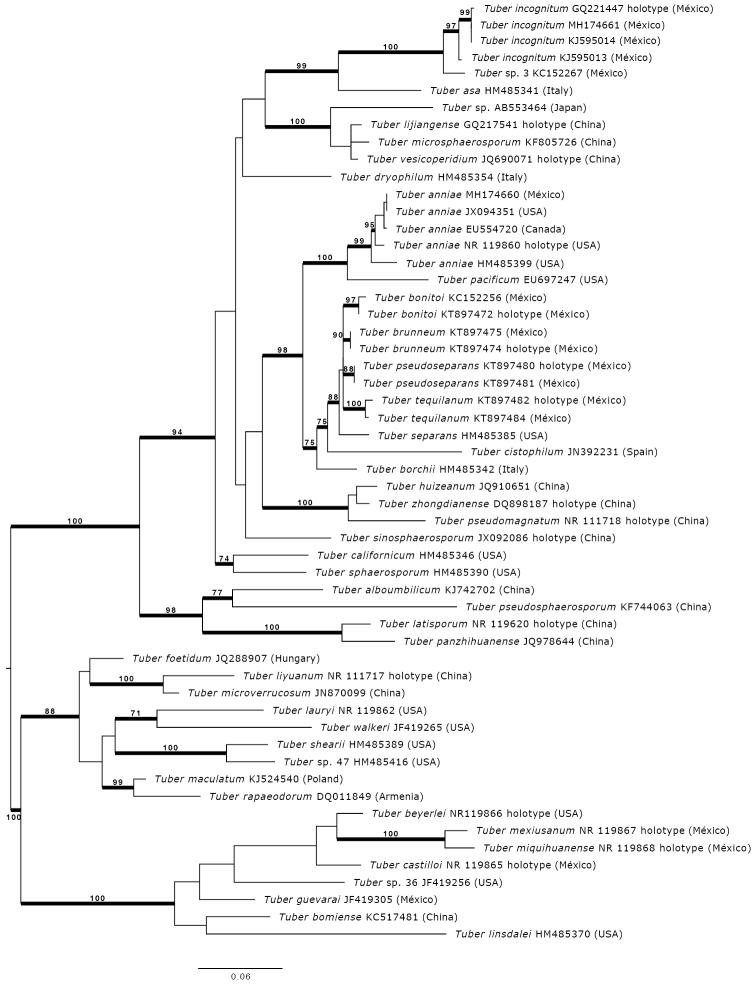
Most likely tree based on maximum likelihood phylogenetic inference showing the placement of *Tuberincognitum* within the Puberulum clade. Bootstrap values ≥ 70% are labelled above nodes. Nodes with posterior probabilities ≥ 99% are blacked. Holotype collections are labelled. The phylogeny is rooted with species belonging to the Maculatum clade. Scale bar corresponds to the mean number of nucleotides substitutions per site.

Species in Puberulum clade can be found in North America, Europe and Asia and some regions of North Africa and South America (Bonito et al. 2010, 2013; Jeandroz et al. 2008; Payen et al. 2014). There are some records of species in the Puberulum clade (e.g. *Tuberrapaeodorum*) that have been introduced into Australia and New Zealand (Bonito et al. 2010). Species in this clade show a wider range of host associations than other species within the Rufum, Excavatum, Aestivum, Maculatum and Gennadii clades (Payen et al. 2014). Species within the Puberulum clade commonly form associations with angiosperms and conifers (Bidartondo et al. 2004; Bonito et al. 2010). In México, twenty species of *Tuber* have been reported, including the two species from this study. Eight of the twenty belong to the Puberulum clade. Both the Maximum Likelihood and Bayesian analyses (Figure 3) show that *T.incognitum* forms a strongly supported clade (Maximum Likelihood bootstrap= 97), which includes sequences from both voucher collections and EcM root tip collections. This clade is placed as a sister taxon of the undescribed species *Tuber* sp. 3 (KC152267) also from México. This study combines sporocarp anatomy, molecular analyses and phylogenetic analyses to support the erection of *T.incognitum* as a unique species within the Puberulum clade.

The *T.anniae* species complex is recovered as a strongly supported clade. There is an internal structure in this clade, with different branch lengths and nested subclades, but additional markers are needed to resolve relationships within this species complex. The Mexican specimens’ group with those from Alaska (JX094351), form a nested clade that is closely related to a collection from Canada (EU554720). The members in the *T.anniae* species complex are closely related to *T.pacificum* from Oregon, USA. Given the relatively high ITS similarity, phylogenetic position and similar morphology to the *T.anniae* holotype collection, we have identified the Mexican collection as *T.anniae*, extending its known range and southernmost distribution of this species in North America.

## Supplementary Material

XML Treatment for Tuber incognitum

XML Treatment for Tuber anniae
